# A One-Pot Six-Component Reaction for the Synthesis of 1,5-Disubstituted Tetrazol-1,2,3-Triazole Hybrids and Their Cytotoxic Activity against the MCF-7 Cell Line

**DOI:** 10.3390/molecules26206104

**Published:** 2021-10-10

**Authors:** Cesia M. Aguilar-Morales, Jorge Gustavo Araujo-Huitrado, Yamilé López-Hernández, Claudia Contreras-Celedón, Alejandro Islas-Jácome, Angelica Judith Granados-López, Cesar R. Solorio-Alvarado, Jesús Adrián López, Luis Chacón-García, Carlos J. Cortés-García

**Affiliations:** 1 Laboratorio de Diseño Molecular, Instituto de Investigaciones Químico-Biológicas, Universidad Michoacana de San Nicolás de Hidalgo, Ciudad Universitaria, Morelia C.P. 58033, Mexico; cesia_aguilar@hotmail.com (C.M.A.-M.); claudia.contreras@umich.mx (C.C.-C.); 2 Laboratorio de microRNAs y Cáncer, Universidad Autónoma de Zacatecas, Av. Preparatoria S/N, Agronómica, Campus II, Zacatecas C.P. 98066, Mexico; aahj011871@uaz.edu.mx (J.G.A.-H.); agranadosjudith@gmail.com (A.J.G.-L.); jalopez@uaz.edu.mx (J.A.L.); 3 Laboratorio de Metabolómica y Proteómica, CONACyT-Universidad Autónoma de Zacatecas, Av. Preparatoria S/N, Agronómica, Campus II, Zacatecas C.P. 98066, Mexico; ylopezher@conacyt.mx; 4 Departamento de Química, Universidad Autónoma Metropolitana-Iztapalapa, San Rafael Atlixco 186, Col. Vicentina, Ciudad de Mexico C.P. 09340, Mexico; aij@xanum.uam.mx; 5 Departamento de Química, División de Ciencias Naturales y Exactas, Universidad de Guanajuato, Campus Guanajuato, Noria Alta S/N, Guanajuato C.P. 36050, Mexico; csolorio@ugto.mx

**Keywords:** Ugi-azide, high-order multicomponent reaction, 1,5-disubstituted tetrazole, 1,4-disubstituted-1,2,3-triazoles, hybrid compounds, cytotoxic activity

## Abstract

A high-order multicomponent reaction involving a six-component reaction to obtain the novel linked 1,5-disubstituted tetrazole-1,2,3-triazole hybrids in low to moderate yield is described. This one-pot reaction is carried out under a cascade process consisting of three sequential reactions: Ugi-azide, bimolecular nucleophilic substitution (S_N_2), and copper-catalyzed alkyne–azide reaction (CuAAC), with high atom and step-economy due the formation of six new bonds (one C-C, four C-N, and one N-N). Thus, the protocol developed offers operational simplicity, mild reaction conditions, and structural diversity. Finally, to evaluate the antitumoral potential of the synthetized molecules, a proliferation study was performed in the breast cancer (BC) derived cell line MCF-7. The hybrid compounds showed several degrees of cell proliferation inhibition with a remarkable effect in those compounds with cyclohexane and halogens in their structures. These compounds represent potential drug candidates for breast cancer treatment. However, additionally assays are needed to elucidate their complete effect over the cellular hallmarks of cancer.

## 1. Introduction

Five-membered aromatic nitrogenous heterocyclic systems are mostly considered privileged structures in medicinal chemistry and are widely used in the development of synthetic strategies to obtain complex and structurally diverse libraries of bioactive compounds [1,2,3,4,5], being a topical challenge for organic chemists [6,7,8]. In recent years, 1,5-disubstituted tetrazoles (1,5-DS-T) and 1,2,3-triazoles have become a starting point in drug discovery projects due to their remarkable biological activities and their presence in marketed drugs, such as cephalosporin-like antibiotics, cilostazol, tazobactam, ticagrelor, and rufinamide [9,10,11,12,13,14]. In parallel, medicinal chemistry has developed molecular hybridization as a promising strategy to achieve highly active compounds with good selectivity and a desirable pharmacokinetic profile. The binding of at least two bioactive motifs (pharmacophores) or privileged structures in a single molecule is the essence of molecular hybridization [15,16,17].

**Figure 1:**
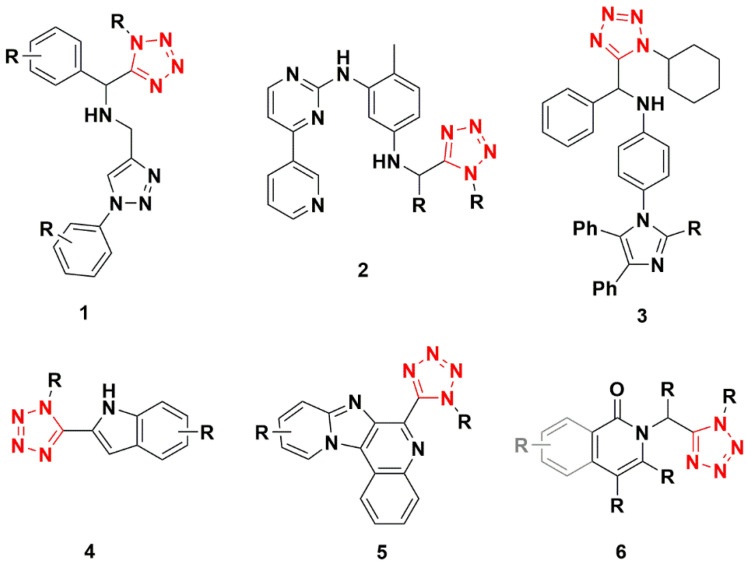
Examples of some hybrids systems containing a 1,5-DS-T moiety via Ugi-azide reaction.

Thus, heterocyclic compounds, such as 1,5-DS-T or 1,2,3-triazoles, and molecular hybridization make a powerful combination in the generation of libraries of compounds with potential biological activity [12,18,19,20,21]. This synergy is even more favored if efficient synthetic strategies such as isocyanide-based multicomponent reactions (I-MCR) are applied [22,23,24]. Among this group of MCRs, the Ugi-azide reaction undoubtedly occupies a relevant place in the synthetic landscape where the generation of 1,5-DS-T libraries becomes promising. It allows the fast, diverse, and efficient generation of series of compounds, which is further demonstrated by the synthesis of various hybrid systems containing 1,5-DS-T moiety, such as triazole-tetrazole **1** [25], phenylamino pyrimidine-tetrazole **2** [26], imidazole-tetrazole (**3**) [27], indole-tetrazole **4** [28], imidazol [1,2-a]pyridine-tetrazole **5** [29], and isoquinolone/pyridone-tetrazole **6** [30] (Figure 1).

In a previous work, we described a synthetic strategy for obtaining a series of 1,5-DS-T hybrid compounds linked to 1,2,3-triazoles in a two-step reaction process by the Ugi-azide reaction, followed by copper-catalyzed azide cycloaddition (CuAAC) as a post-condensation reaction, describing a reaction of its type for the first time in an Ugi-azide process [25]. Inspired by these results, we now decided to obtain the hybrid system in a one-pot process by a high-order multicomponent reaction. This is scarcely explored in comparison to a four- or three-component reaction [31]. Therefore, herein, we report a new high-order multicomponent reaction to synthetize novel 1,5-disubstituted tetrazole-1,2,3-triazoles hybrid system involving a six-component reaction under a cascade process (Ugi-azide, bimolecular nucleophilic substitution (S_N_2), and CuAAC). In addition, to evaluate the biological potential of these molecules, we also report the proliferation inhibition of the MCF-7 cell line, a model for breast cancer (BC). With 684,996 deaths registered annually [32], BC is one of most frequent carcinomas and deathliest pathologies worldwide [33,34].

## 2. Results and Discussion

In order to find the optimal reaction conditions for the high-order multicomponent reaction, we started with the synthesis of **13a** as a model reaction using 2-fluorobenzaldehyde (**8**), azidotrimethylsilane (**9**), *tert*-butyl isocyanide (**10**), sodium azide (**11**), benzyl bromide (**12**), and propargylamine (**7**), the latter being the orthogonal starting material, which acts as a bifunctional reagent containing both the primary amino group for the initial MCR, and the alkyne group for the CuAAC reaction (Table 1). In a first attempt, the classical Ugi-azide reaction conditions were used, i.e., MeOH (1M) at room temperature, yielding the Ugi-azide product within two hours by monitoring the reaction by TLC. It is worth mentioning that formation of this product was reported by us [25], and in the present work, its isolation was not necessary, because the synthesis was performed in a one-pot fashion. The next step was the addition of sodium azide (**11**) and benzyl bromide (**12**) to the reaction mixture. The reaction was kept under stirring to finally add CuSO_4_^.^5H_2_O and sodium ascorbate to obtain the product **13a** in 24 h, yielding 58% after purification (entry 1). The change of the solvent to glycerol, considering Zhao’s report, in which 1,4-disubstituted 1,2,3-triazoles were efficiently synthesized [35], showed a considerable decrease in the reaction yield to 11%, with considerable recovery of starting materials (entry 2). The drawback appeared to be more mechanical than chemical, as the reaction mixture was too dense and did not facilitate proper stirring. The same technical problem, as well as low yield (15%), was observed when *t*-BuOH:H_2_O was used as a solvent system [25]. (Entry 3) Finally, although very efficient for the synthesis of 1,5-DS-tetrazole and S_N_2, 2,2,2-trifluoroethanol (TFE) did not yield the expected product in the CuAAC reaction (Entry 4).

**Scheme 1:**
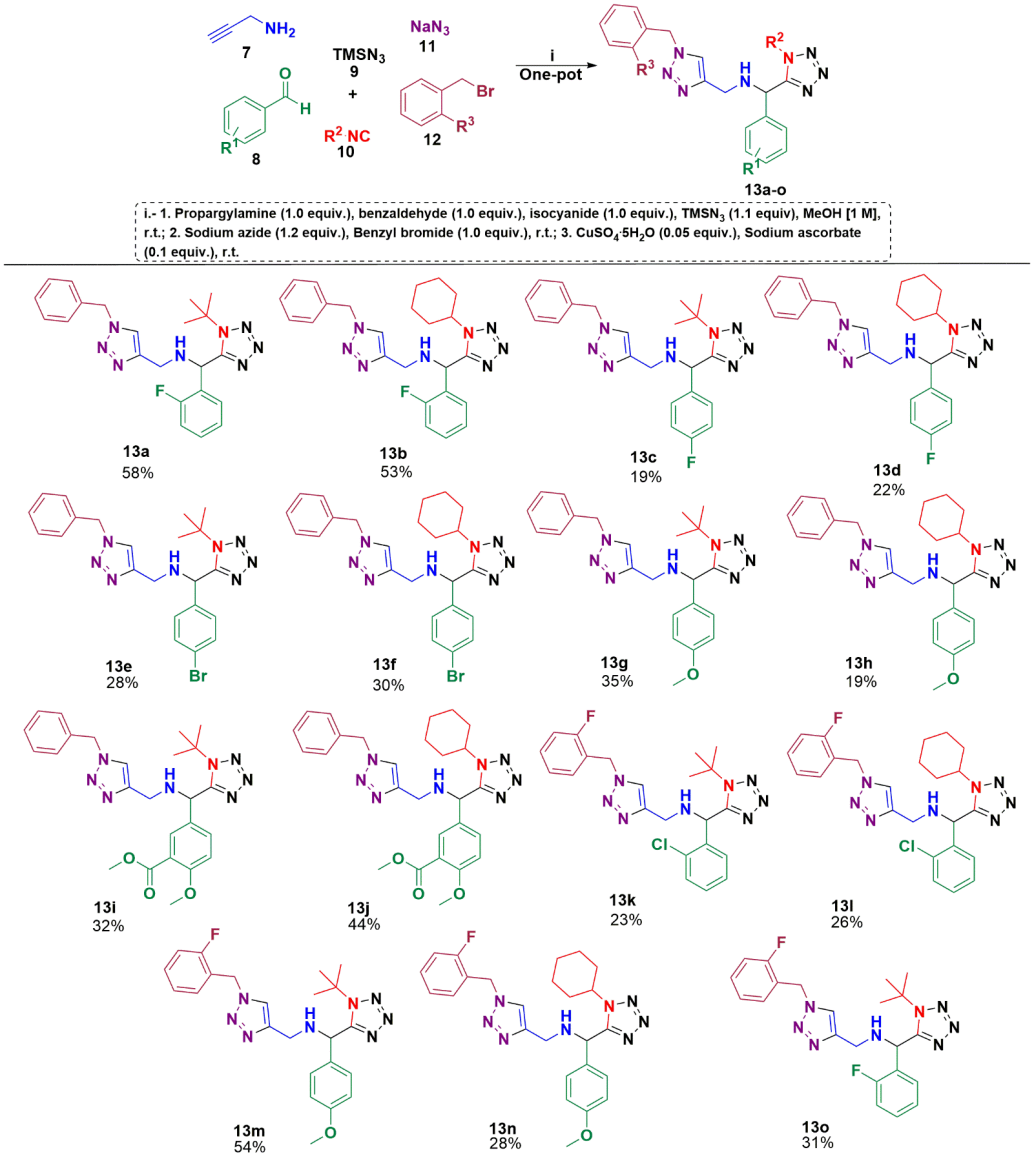
One-pot six-component synthesis of 1,5-disubstituted tetrazole-1,2,3-triazole hybrids.

Once the optimal reaction conditions were found, the scope of the procedure was evaluated to generate a new library of hybrid compounds of 1,5-disubstituted tetrazol-1,2,3-triazoles, decorated with groups of different stereoelectronic nature. Thus, the diversity of the reaction can be seen in the Scheme 1, obtaining the target molecules **13a–o** in 22–58% yields. The identity of all compounds was confirmed by ^1^H and ^13^C NMR spectroscopy as well as HRMS. As it can be seen, the best yields were obtained using *tert*-butyl isocyanide. With respect to the influence of substituents from benzaldehyde component, electron-withdrawing groups such as fluorine in the *ortho* position and electron-donating groups such as methoxy in *para* position gave the best yields. On the other hand, the lowest yields were observed when benzaldehyde fluorinated in *para* position was used.

**Scheme 2:**
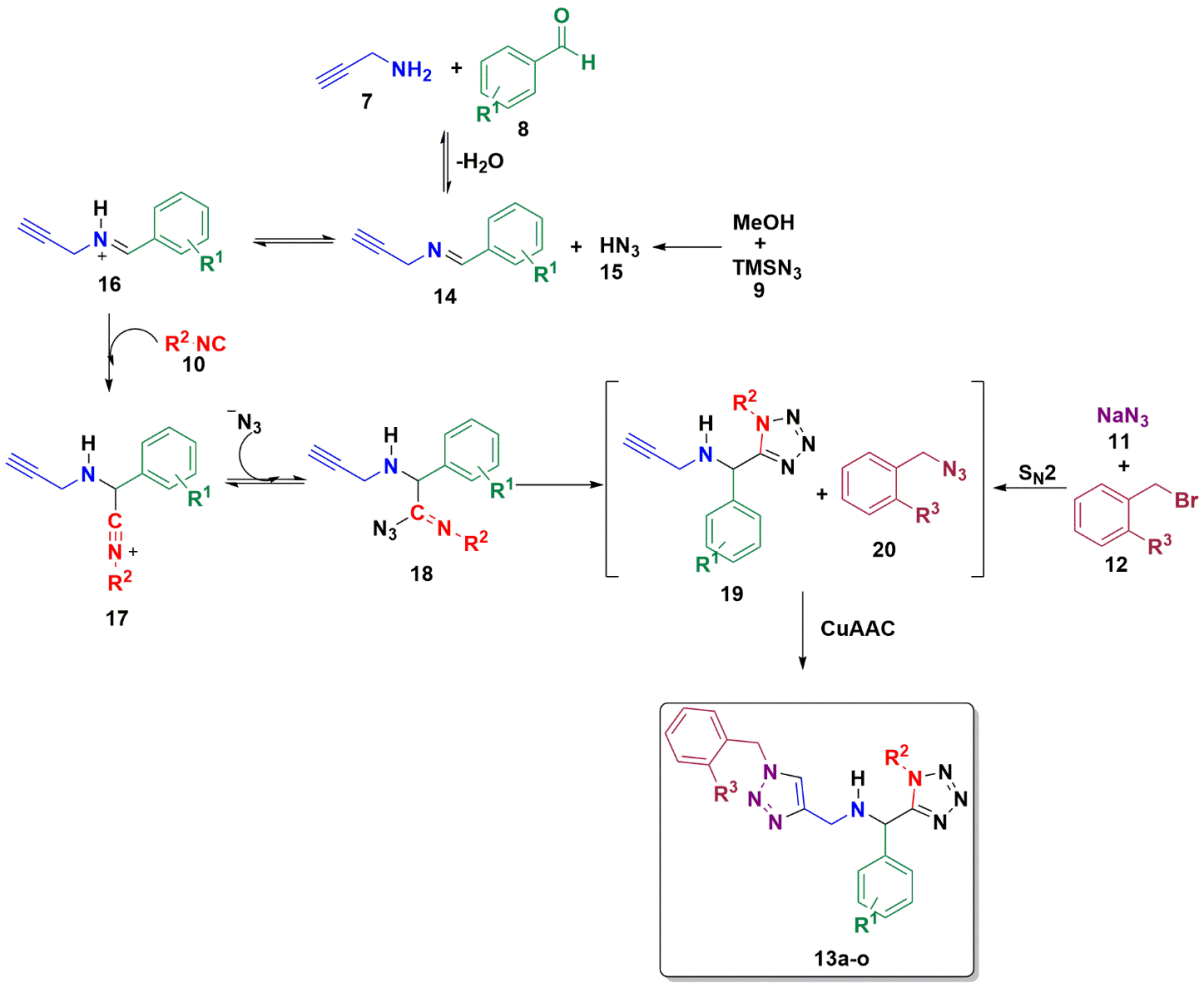
Cascade process for the synthesis of target molecules **13a–o**.

The cascade reaction mechanism behind the target molecules **13a–o** is depicted in Scheme 2. First, propargylamine **7** reacts with benzaldehyde derivate **8** to give the imine **14**, which is protonated with HN_3_ (generated in situ by proton exchange between TMSN_3_ and methanol) affording the iminium ion **16**. This ion is trapped by the isocyanide **10** to give the nitrilium ion **17**, which is then attacked by the azide ion to afford the 1,5-DS-T **19** after of spontaneous 1,5-electrocyclization of **18**. On the other hand, benzyl azide **20** is formed in situ via bimolecular nucleophilic substitution between sodium azide and benzyl bromide **12**. Finally, a copper catalyzed alkyne–azide reaction takes place by reacting 1,5-DS-T **19** with benzyl azide **20** to afford the 1,5-disubstituted tetrazole-1,2,3-triazole hybrids **13**.

**Figure 2:**
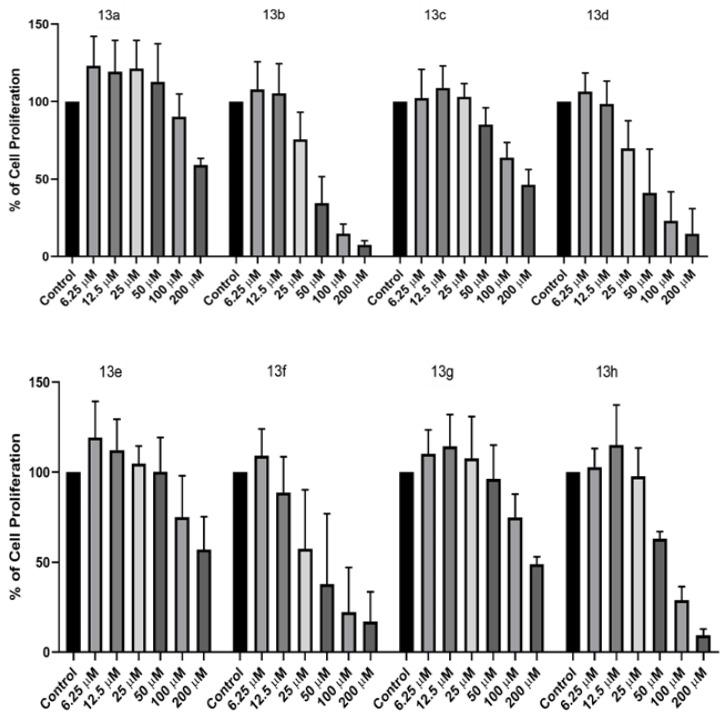

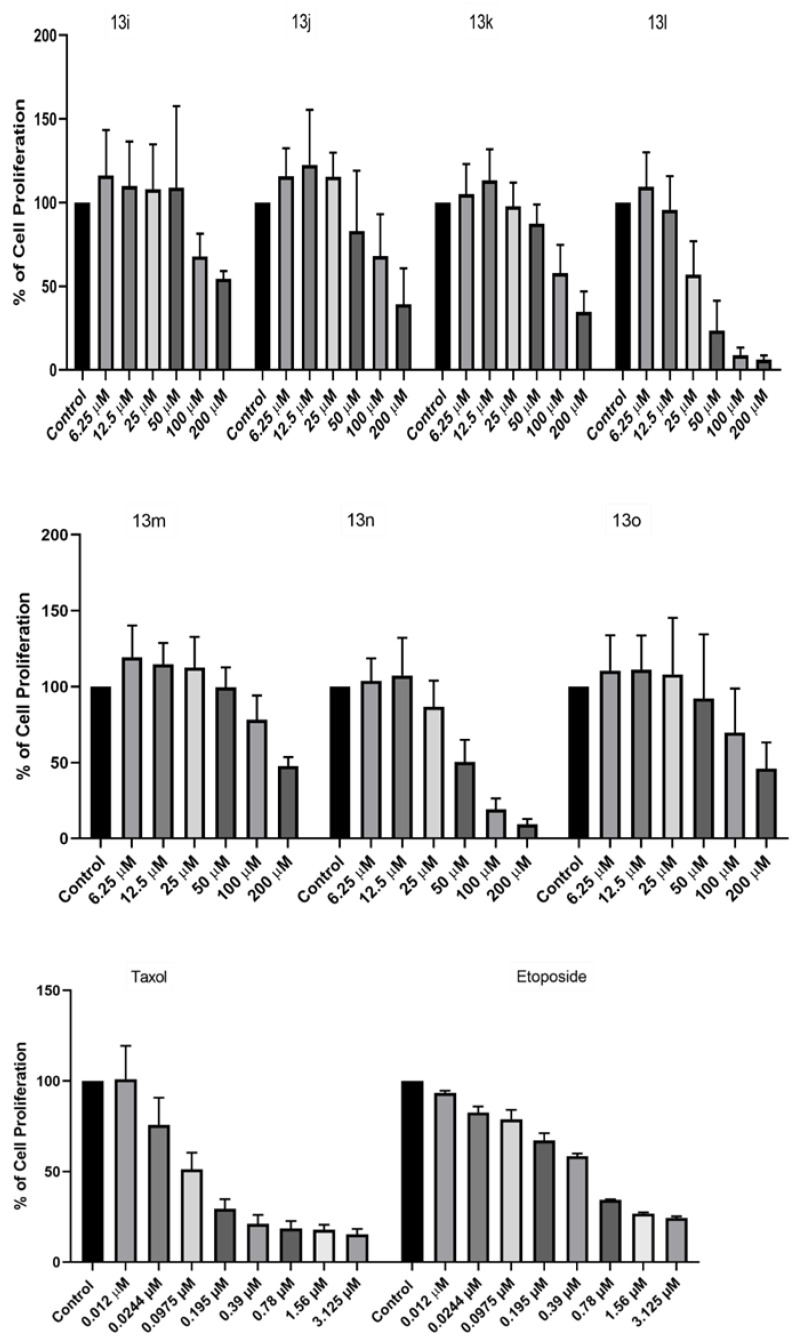
Differential effect of the compounds **13a–o** in the proliferation of MCF-7 cell line. MCF-7 cells were treated with increased doses of compounds **13a** to **13o** and Taxol and Etoposide were used to compare the effect. Control cells are cells not treated.

Compounds **13a–o** were tested to evaluate their capacity to inhibit cell proliferation as one of the principal hallmarks of cancer. The cell line MCF-7 with a genotype ER+, PR+, or HER2− is a model of invasive breast ductal carcinoma, representing 70 to 80% of invasive BC. Cell proliferation inhibition of MCF-7 cells was primarily achieved by the following compounds in the next order: **13f**, **13l**, **13b**, **13d**, **13h**, **13k**, **13j**, **13n**, **13o**, **13a**, **13g**, **13e**, **13i**, **13m**, and **13c** (Figure 2). Taxol and Etoposide were used as chemical controls of cell death induction (Figure 2 and Table 1).

A noticeable effect difference of near 200 and 25 times was observed in compound **13f** (19.76) versus Taxol and Etoposide (0.0975 and 0.78 µM), respectively. However, this difference must by enclosed in the resistance that tumors present [36]. The combination of alternative compounds could be used for tumor cells with a resistance to Taxol and/or Etoposide treatments [37]. The activity of these compounds is not outstanding, but it is comparable with other groups of compounds, such as those reported by Shaaban and co-workers. Shaaban et al. reported 18 novel tetrazoles-based diselenides and seleoquinones, which were synthetized via an Ugi-azide reaction and assayed in MCF-7 cell lines, and the proliferation inhibition was ranked from 14 to 78 µM [38]. Our results are also comparable to those found by Nagarapu research group, in which the synthesis of a series of 1,2,3-triazole tethered chalcone acetamides was reported, ranking from an IC_50_ of 9.76 to 98 µM [39]. Additionally, carboxyamido-triazoles showed an IC_50_ of 16 µM on MCF-7 cells compared with **13f** compound, which showed an IC_50_ of 19.76 µM. It should be considered that carboxyamido-triazoles have been tested in vivo in humans [40]. In this context, modifications to the structure of compound **13f** are underway to achieve better results in proliferation inhibition as well as other hallmarks of cancer.

Finally, the cytotoxic activity assays IC_50_(µM) summarized in Table 2 indicate that growth inhibition is mainly influenced by the tetrazole substituent, where all compounds containing cyclohexyl (compounds **13b**, **13d**, **13f**, **13h**, **13j**, **13l**, and **13n**) are more active, whereas the *tert*-butyl group leads to a loss of activity, with the exception of compounds **13e** and **13k**, where halogen (chlorine or bromine) probably plays an important role, as they also do in the cyclohexyl derivatives **13f** and **13l**, which are the most active of the whole series of compounds. On the other hand, the replacement of hydrogen by fluorine in the benzyl moiety notoriously favored an increase in the activity, as can be seen when comparing compounds **13h** and **13n**, where the presence of a fluorine atom in the position 2 gives rise to a change in the IC_50_ from 41.67 to 29.25 µM.

## 3. Materials and Methods

### 3.1. Chemistry

#### 3.1.1. General Information

All reagents, reactants, and solvents were purchased from Merck (Darmstad, Germany) without further purification. Melting points (uncorrected) were determined on a Fisher-Johns melting point apparatus. Column chromatography was performed using silica gel (230–400 mesh). Reaction progress was monitored by thin layer chromatography (TLC) with silica gel plates from Merck (silica gel 60 F_254_), and the spots were visualized under UV light at 254 or 365 nm. Chemical names and drawings were obtained using ChemDraw Professional (version 18.0.0.231). NMR spectra were recorded on a Varian Mercury (400 Mhz) spectrometer, using CDCl_3_ as the solvent and TMS as the internal reference. Chemical shifts were reported as δ values (ppm). Coupling constants *J* are reported in Hertz (Hz). High-resolution mass spectra (HRMS) were performed on Brucker microTOF.

#### 3.1.2. General Procedure for Compounds **13a–o** (GP)

Propargylamine (1.0 equiv.) and aldehyde (1.0 equiv.) were dissolved in MeOH (1 M) and reacted for 5 min; then, isocyanide (1.0 equiv.) and TMSN_3_ (1.2 equiv.) were sequentially added and stirred at room temperature for 2 h. Next, benzyl bromide (1.0 equiv.) and NaN_3_ (1.2 equiv.) were added, and the reaction mixture was stirred for 2 h at room temperature. Then, CuSO_4_^.^5H_2_O (0.05 equiv.) and sodium ascorbate (0.10 equiv.) were added. The reaction mixture was stirred for 24 h at room temperature. After, water (10 mL) and ethyl acetate (5 mL) were added, and the layers were separated. Aqueous layer was extracted with EtOAc (5 mL). The organic phases were washed twice with water (5 mL), dried over anhydrous Na_2_SO_4_, and evaporated under vacuum. The reaction crude was purified by flash column chromatography with Hexane:EtOAc 4:6 (*v/v*) to afford the compounds **13a–o**. 

#### 3.1.3. Synthesis and Characterization of Compounds **13a–o**

*N-((1-benzyl-1H-1,2,3-triazol-4-yl)methyl)-1-(1-(tert-butyl)-1H-tetrazol-5-yl)-1-(2-fluorophenyl)methanamine* (**13a**). Based on the GP1, propargylamine (23.2 µL, 0.36 mmol), 2-fluorobenzaldehyde (38.2 µL, 0.36 mmol), TMSN_3_ (57.2 µL, 0.43 mmol), *tert*-butyl isocyanide (41.0 µL, 0.36 mmol), benzyl bromide (43.1 µL, 0.36 mmol), sodium azide (28.2 mg, 0.43 mmol), CuSO_4_^.^5H_2_O (4.0 mg, 0.016 mmol), and sodium ascorbate (6.4 mg, 0.032 mmol) were used, and 13a was obtained as a yellow oil (yield 88.5 mg, 58%). R*_f_* = 0.51 (Hexane-AcOEt 4:6 *v/v*); ^1^H NMR (400 MHz, CDCl_3_): **δ** = 7.46 (s, 1H), 7.40–7.35 (m, 3H), 7.31–7.24 (m, 4H), 7.12–7.05 (m, 2H), 5.78 (s, 1H), 5.51 (s, 2H), 3.90 (q, *J* = 12.0 Hz, 2H), 1.55 (s, 9H). ^13^C NMR (100 MHz, CDCl_3_): **δ** = 159.8 (d, *J* = 246.9 Hz), 154.6, 146.3, 134.6, 130.3 (d, *J* = 8.3 Hz), 129.0, 128.9 (d, *J* = 2.9 Hz), 128.7, 128.0, 125.7 (d, *J* = 14.0 Hz), 125.0 (d, *J* = 3.5 Hz), 121.9, 115.7 (d, *J* = 22.1 Hz), 61.6, 54.1, 49.6, 42.7, 29.6 (Please see Supplementary Materials). HRMS (ESI^+^): m/z: Calcd. for C_22_H_26_FN_8_ [M + H]^+^: 421.2264; Found: 421.2268.

*N-((1-benzyl-1H-1,2,3-triazol-4-yl)methyl)-1-(1-cyclohexyl-1H-tetrazol-5-yl)-1-(2-fluorophenyl)methanamine* (**13b**). Based on the GP1, propargylamine (23.2 µL, 0.36 mmol), 2-fluorobenzaldehyde (38.2 µL, 0.36 mmol), TMSN_3_ (57.2 µL, 0.43 mmol), cyclohexyl isocyanide (45.1 µL, 0.36 mmol), benzyl bromide (43.1 µL, 0.36 mmol), sodium azide (28.2 mg, 0.43 mmol), CuSO_4_^.^5H_2_O (4.0 mg, 0.016 mmol), and sodium ascorbate (6.4 mg, 0.032 mmol) were used, and 13b was obtained as a beige solid (yield 85.6 mg, 53%). m.p. = 76–78 °C, R*_f_* = 0.40 (Hexane-AcOEt 4:6 *v/v*); ^1^H NMR (400 MHz, CDCl_3_): **δ** = 7.41–7.36 (m, 5H), 7.31–7.25 (m, 3H), 7.17–7.13 (m, 1H), 7.09–7.04 (m, 1H), 5.57 (s, 1H), 5.51 (s, 2H), 4.22–4.15 (m, 1H), 3.90 (q, *J* = 12.0 Hz, 2H), 1.99–1.21 (m, 10H). ^13^C NMR (100 MHz, CDCl_3_): **δ** = 158.9, 154.1, 145.9, 134.5, 130.4 (d, *J* = 8.5 Hz), 129.1, 128.8, 128.7, 128.1, 125.1 (d, *J* = 3.5 Hz), 122.0, 115.7 (d, *J* = 22.0 Hz), 57.9, 54.1, 48.8, 42.3, 32.7, 32.6, 25.2, 25.2, 24.7. HRMS (ESI^+^): m/z: Calcd. for C_24_H_28_FN_8_ [M + H]^+^: 447.2421; Found: 447.2421.

*N-((1-benzyl-1H-1,2,3-triazol-4-yl)methyl)-1-(1-(tert-butyl)-1H-tetrazol-5-yl)-1-(4-fluorophenyl)methanamine* (**13c**). Based on the GP1, propargylamine (23.2 µL, 0.36 mmol), 4-fluorobenzaldehyde (38.9 µL, 0.36 mmol), TMSN_3_ (57.2 µL, 0.43 mmol), *tert*-butyl isocyanide (41.0 µL, 0.36 mmol), benzyl bromide (43.1 µL, 0.36 mmol), sodium azide (28.2 mg, 0.43 mmol), CuSO_4_^.^5H_2_O (4.0 mg, 0.016 mmol), and sodium ascorbate (6.4 mg, 0.032 mmol) were used, and **13c** was obtained as a beige solid (yield 29.3 mg, 19%). m.p. = 113–115 °C, R*_f_* = 0.53 (Hexane-AcOEt 4:6 *v/v*); ^1^H NMR (400 MHz, CDCl_3_): **δ** = 7.40 (s, 1H), 7.38–7.36 (m, 3H), 7.29–7.25 (m, 4H), 7.03–6.98 (m, 2H), 5.50 (s, 3H), 3.83 (q, *J* = 14.0 Hz, 2H), 1.96 (s, 9H). ^13^C NMR (100 MHz, CDCl_3_): **δ** = 162.5 (d, *J* = 248.0 Hz), 155.2, 146.5, 134.5, 134.3, 130.0 (d, *J* = 8.3 Hz), 129.1, 128.8, 128.1, 121.9, 115.9 (d, *J* = 21.5 Hz), 61.5, 56.4, 54.2, 42.1, 29.9. HRMS (ESI^+^): m/z: Calcd. for C_22_H_26_FN_8_ [M + H]^+^: 421.2264; Found: 421.2277.

*N-((1-benzyl-1H-1,2,3-triazol-4-yl)methyl)-1-(1-cyclohexyl-1H-tetrazol-5-yl)-1-(4-fluorophenyl)methanamine* (**13d**). Based on the GP1, propargylamine (23.2 µL, 0.36 mmol), 4-fluorobenzaldehyde (38.9 µL, 0.36 mmol), TMSN_3_ (57.2 µL, 0.43 mmol), cyclohexyl isocyanide (45.1 µL, 0.36 mmol), benzyl bromide (43.1 µL, 0.36 mmol), sodium azide (28.2 mg, 0.43 mmol), CuSO_4_^.^5H_2_O (4.0 mg, 0.016 mmol), and sodium ascorbate (6.4 mg, 0.032 mmol) were used, and 13d was obtained as a beige solid (yield 36.0 mg, 22%). m.p. = 83–85 °C, R*_f_* = 0.53 (Hexane-AcOEt 4:6 *v/v*); ^1^H NMR (400 MHz, CDCl_3_): δ = 7.39–7.31 (m, 6H), 7.28–7.26 (m, 2H), 7.05–7.01 (m, 2H), 5.55–5.46 (m, 2H), 5.34 (s, 1H), 4.24–4.17 (m, 1H), 3.83 (q, *J* = 16 Hz, 2H), 2.40 (bs, 1H), 1.90–1.19 (m, 10H). ^13^C NMR (100 MHz, CDCl_3_): δ = 162.6 (d, *J* = 248.2 Hz), 154.3, 145.8, 134.4, 133.6 (d, *J* = 3.3 Hz), 129.3 (d, *J* = 8.2 Hz), 129.1, 128.8, 128.1, 122.0, 116.0 (d, *J* = 21.7 Hz), 58.0, 55.4, 54.2, 41.9, 32.6, 32.5, 25.2, 25.2, 24.7. HRMS (ESI^+^): m/z: Calcd. for C_24_H_28_FN_8_ [M + H]^+^: 447.2421; Found: 447.2441.

*N-((1-benzyl-1H-1,2,3-triazol-4-yl)methyl)-1-(4-bromophenyl)-1-(1-(tert-butyl)-1H-tetrazol-5-yl)methanamine* (**13e**). Based on the GP1, propargylamine (23.2 µL, 0.36 mmol), 4-bromobenzaldehyde (67.1 mg, 0.36 mmol), TMSN_3_ (57.2 µL, 0.43 mmol), *tert*-butyl isocyanide (41.0 µL, 0.36 mmol), benzyl bromide (43.1 µL, 0.36 mmol), sodium azide (28.2 mg, 0.43 mmol), CuSO_4_^.^5H_2_O (4.0 mg, 0.016 mmol), and sodium ascorbate (6.4 mg, 0.032 mmol) were used, and 13e was obtained as a white solid (yield 48.6 mg, 28%). m.p. = 113–115 °C, R*_f_* = 0.48 (Hexane-AcOEt 4:6 *v/v*); ^1^H NMR (400 MHz, CDCl_3_): δ = 7.45 (d, *J* = 8.4 Hz, 2H), 7.39–7.36 (m, 4H), 7.28–7.26 (m, 2H), 7.17 (d, *J* = 8.5 Hz, 2H), 5.50 (s, 2H), 5.48 (s, 1H), 3.83 (q, *J* = 14.0 Hz, 2H), 1.56 (s, 9H). ^13^C NMR (100 MHz, CDCl_3_): δ = 155.0, 146.4, 137.4, 134.5, 132.1, 129.9, 129.1, 128.8, 128.1, 122.5, 121.9, 61.5, 56.5, 54.2, 42.1, 29.9. HRMS (ESI^+^): m/z: Calcd. for C_22_H_26_BrN_8_ [M + H]^+^: 481.1464; Found: 481.1467. 

*N-((1-benzyl-1H-1,2,3-triazol-4-yl)methyl)-1-(4-bromophenyl)-1-(1-cyclohexyl-1H-tetrazol-5-yl)methanamine* (**13f**). Based on the GP1, propargylamine (23.2 µL, 0.36 mmol), 4-bromobenzaldehyde (67.1 mg, 0.36 mmol), TMSN_3_ (57.2 µL, 0.43 mmol), cyclohexyl isocyanide (45.1 µL, 0.36 mmol), benzyl bromide (43.1 µL, 0.36 mmol), sodium azide (28.2 mg, 0.43 mmol), CuSO_4_^.^5H_2_O (4.0 mg, 0.016 mmol), and sodium ascorbate (6.4 mg, 0.032 mmol) were used, and 13f was obtained as a beige solid (yield 56.8 mg, 30%). m.p. = 101–103 °C, R*_f_* = 0.51 (Hexane-AcOEt 4:6 *v/v*); ^1^H NMR (400 MHz, CDCl_3_): δ = 7.47 (d, *J* = 8.3 Hz, 2H), 7.38–7.37 (m, 4H), 7.28-7.26 (m, 2H), 7.24 (d, *J* = 8.4 Hz, 2H), 5.50 (d, *J* = 4.0 Hz, 2H), 5.31 (s, 1H), 4.25–4.18 (m, 1H), 3.83 (q, *J* = 17.3 Hz, 2H), 1.89–1.21 (m, 10H). ^13^C NMR (100 MHz, CDCl_3_): δ = 154.0, 145.7, 136.8, 134.4, 132.1, 129.2, 129.1, 128.8, 128.1, 122.6, 122.0, 58.0, 55.4, 54.1, 41.9, 32.6, 32.5, 25.2, 25.2, 24.6. HRMS (ESI^+^): m/z: Calcd. for C_24_H_28_BrN_8_ [M + H]^+^: 507.1620; Found: 507.1630.

*N-((1-benzyl-1H-1,2,3-triazol-4-yl)methyl)-1-(1-(tert-butyl)-1H-tetrazol-5-yl)-1-(4-methoxyphenyl)methanamine* (**13g**). Based on the GP1, propargylamine (23.2 µL, 0.36 mmol), 4-methoxybenzaldehyde (44.1 µL, 0.36 mmol), TMSN_3_ (57.2 µL, 0.43 mmol), *tert*-butyl isocyanide (41.0 µL, 0.36 mmol), benzyl bromide (43.1 µL, 0.36 mmol), sodium azide (28.2 mg, 0.43 mmol), CuSO_4_^.^5H_2_O (4.0 mg, 0.016 mmol), and sodium ascorbate (6.4 mg, 0.032 mmol) were used, and 13g was obtained as a yellow oil (yield 55.3 mg, 35%). R*_f_* = 0.37 (Hexane-AcOEt 4:6 *v/v*); ^1^H NMR (400 MHz, CDCl_3_): δ = 7.40 (s, 1H), 7.36–7.34 (m, 3H), 7.26–7.24 (m, 2H), 7.17 (d, *J* = 8.7 Hz, 2H), 6.81 (d, *J* = 8.7 Hz, 2H), 5.48 (s, 2H), 5.40 (s, 1H), 3.87–3.75 (m, 5H), 1.53 (s, 9H). ^13^C NMR (100 MHz, CDCl_3_): δ = 159.4, 155.5, 146.7, 134.5, 130.3, 129.4, 129.0, 128.7, 128.1, 121.9, 114.2, 61.4, 56.6, 55.2, 54.1, 42.2, 29.8. HRMS (ESI^+^): m/z: Calcd. for C_23_H_29_N_8_O [M + H]^+^: 433.2464; Found: 433.2475.

*N-((1-benzyl-1H-1,2,3-triazol-4-yl)methyl)-1-(1-cyclohexyl-1H-tetrazol-5-yl)-1-(4-methoxyphenyl)methanamine* (**13h**). Based on the GP1, propargylamine (23.2 µL, 0.36 mmol), 4-methoxybenzaldehyde (44.1 µL, 0.36 mmol), TMSN_3_ (57.2 µL, 0.43 mmol), cyclohexyl isocyanide (45.1 µL, 0.36 mmol), (41.0 µL, 0.36 mmol), benzyl bromide (43.1 µL, 0.36 mmol), sodium azide (28.2 mg, 0.43 mmol), CuSO_4_^.^5H_2_O (4.0 mg, 0.016 mmol), and sodium ascorbate (6.4 mg, 0.032 mmol) were used, and 13h was obtained as a beige solid (yield 31.4 mg, 19%). m.p. = 73–75 °C, R*_f_* = 0.42 (Hexane-AcOEt 4:6 *v/v*); ^1^H NMR (400 MHz, CDCl_3_): δ = 7.40–7.36 (m, 4H), 7.28–7.26 (m, 3H), 7.24 (d, *J* = 8.7 Hz, 2H), 6.85 (d, *J* = 8.7 Hz, 2H), 5.50 (d, *J* = 4.3 Hz, 2H), 5.25 (s, 1H), 4.23–4.15 (m, 1H), 3.90–3.78 (m, 5H), 1.93–1.17 (m, 10H). ^13^C NMR (100 MHz, CDCl_3_): δ = 159.7, 154.6, 146.1, 134.5, 129.7, 129.1, 128.8, 128.7, 128.1, 122.0, 114.3, 57.9, 55.7, 55.3, 54.2, 42.0, 32.5, 32.4, 25.3, 25.2, 24.7. HRMS (ESI^+^): m/z: Calcd. for C_25_H_31_N_8_O [M + H]^+^: 459.2621; Found: 459.2630.

*methyl 5-((((1-benzyl-1H-1,2,3-triazol-4-yl)methyl)amino)(1-(tert-butyl)-1H-tetrazol-5-yl)methyl)-2-methoxybenzoate* (**13i**). Based on the GP1, propargylamine (23.2 µL, 0.36 mmol), methyl 5-formyl-2-methoxybenzoate (70.4 mg, 0.36 mmol), TMSN_3_ (57.2 µL, 0.43 mmol), *tert*-butyl isocyanide (41.0 µL, 0.36 mmol), benzyl bromide (43.1 µL, 0.36 mmol), sodium azide (28.2 mg, 0.43 mmol), CuSO_4_^.^5H_2_O (4.0 mg, 0.016 mmol), and sodium ascorbate (6.4 mg, 0.032 mmol) were used, and 13i was obtained as a yellow oil (yield 56.5 mg, 32%). R*_f_* = 0.73 (Hexane-AcOEt 4:6 *v/v*); ^1^H NMR (400 MHz, CDCl_3_): δ = 7.74 (d, *J* = 2.4 Hz, 1H), 7.42–7.35 (m, 5H), 7.28–7.26 (m, 2H), 6.92 (d, *J* = 8.7 Hz, 1H), 5.51–5.49 (m, 3H), 3.89–3.81 (m, 8H), 1.57 (s, 9H). ^13^C NMR (100 MHz, CDCl_3_): δ = 166.0, 159.0, 155.1, 146.4, 134.5, 133.2, 131.5, 130.1, 129.1, 128.8, 128.1, 121.9, 120.4, 112.5, 61.5, 56.4, 56.1, 54.2, 52.1, 42.2, 29.9. HRMS (ESI^+^): m/z: Calcd. for C_25_H_31_N_8_O_3_ [M + H]^+^: 491.2519; Found: 491.2531.

*methyl 5-((((1-benzyl-1H-1,2,3-triazol-4-yl)methyl)amino)(1-cyclohexyl-1H-tetrazol-5-yl)methyl)-2-methoxybenzoate* (**13j**). Based on the GP1, propargylamine (23.2 µL, 0.36 mmol), methyl 5-formyl-2-methoxybenzoate (70.4 mg, 0.36 mmol), TMSN_3_ (57.2 µL, 0.43 mmol), cyclohexyl isocyanide (45.1 µL, 0.36 mmol), benzyl bromide (43.1 µL, 0.36 mmol), sodium azide (28.2 mg, 0.43 mmol), CuSO_4_^.^5H_2_O (4.0 mg, 0.016 mmol), and sodium ascorbate (6.4 mg, 0.032 mmol) were used, and 13j was obtained as a white solid (yield 82.3 mg, 44%). m.p. = 110–113 °C, R*_f_* = 0.43 (Hexane-AcOEt 4:6 *v/v*); ^1^H NMR (400 MHz, CDCl_3_): δ = 7.80 (d, *J* = 2.5 Hz, 1H), 7.46 (dd, *J* = 8.7, 2.5 Hz, 1H), 7.39–7.36 (m, 4H), 7.28–7.26 (m, 2H), 6.95 (d, *J* = 8.7 Hz, 1H), 5.51 (d, *J* = 2.1 Hz, 2H), 5.33 (s, 1H), 4.31–4.25 (m, 1H), 3.89–3.77 (m, 8H), 1.90–1.22 (m, 10H). ^13^C NMR (100 MHz, CDCl_3_): δ = 165.9, 159.1, 154.2, 145.8, 134.4, 132.6, 130.6, 129.4, 129.1, 128.8, 128.1, 122.0, 120.3, 112.6, 58.0, 56.1, 55.3, 54.1, 52.1, 41.9, 32.6, 32.5, 25.2, 24.7. HRMS (ESI^+^): m/z: Calcd. for C_27_H_33_N_8_O_3_ [M + H]^+^: 517.2676; Found: 517.2697.

*1-(1-(tert-butyl)-1H-tetrazol-5-yl)-1-(2-chlorophenyl)-N-((1-(2-fluorobenzyl)-1H-1,2,3-triazol-4-yl)methyl)methanamine* (**13k**). Based on the GP1, propargylamine (23.2 µL, 0.36 mmol), 2-chlorobenzaldehyde (40.8 µL, 0.36 mmol), TMSN_3_ (57.2 µL, 0.43 mmol), *tert*-butyl isocyanide (41.0 µL, 0.36 mmol), benzyl bromide (43.1 µL, 0.36 mmol), sodium azide (28.2 mg, 0.43 mmol), CuSO_4_^.^5H_2_O (4.0 mg, 0.016 mmol), and sodium ascorbate (6.4 mg, 0.032 mmol) were used, and 13k was obtained as a beige solid (yield 37.8 mg, 23%). m.p. = 115–117 °C, R*_f_* = 0.47 (Hexane-AcOEt 4:6 *v/v*); ^1^H NMR (400 MHz, CDCl_3_): δ = 7.58 (s, 1H), 7.40–7.35 (m, 2H), 7.30–7.11 (m, 6H), 5.81 (s, 1H), 5.57 (q, *J* = 13.3 Hz, 2H), 3.94 (dd, *J* = 46.0, 14.0 Hz, 2H), 1.54 (s, 9H). ^13^C NMR (100 MHz, CDCl_3_): δ = 160.5 (d, *J* = 248.1 Hz), 154.4, 146.1, 136.3, 133.3, 130.9 (d, *J* = 8.1 Hz), 130.6 (d, *J* = 3.1 Hz), 130.1, 129.7, 128.9, 127.6, 124.8 (d, *J* = 3.8 Hz), 122.3, 121.8 (d, *J* = 14.9 Hz), 115.8 (d, *J* = 21.1 Hz), 61.7, 53.2, 47.6, 42.8, 29.6. HRMS (ESI^+^): m/z: Calcd. for C_22_H_25_ClFN_8_ [M + H]^+^: 455.1875; Found: 455.1886.

*1-(2-chlorophenyl)-1-(1-cyclohexyl-1H-tetrazol-5-yl)-N-((1-(2-fluorobenzyl)-1H-1,2,3-triazol-4-yl)methyl)methanamine* (**13l**). Based on the GP1, propargylamine (23.2 µL, 0.36 mmol), 2-chlorobenzaldehyde (40.8 µL, 0.36 mmol), TMSN_3_ (57.2 µL, 0.43 mmol), cyclohexyl isocyanide (45.1 µL, 0.36 mmol), benzyl bromide (43.1 µL, 0.36 mmol), sodium azide (28.2 mg, 0.43 mmol), CuSO_4_^.^5H_2_O (4.0 mg, 0.016 mmol), and sodium ascorbate (6.4 mg, 0.032 mmol) were used, and 13l was obtained as a yellow oil (yield 45.2 mg, 26%). R*_f_* = 0.47 (Hexane-AcOEt 4:6 *v/v*); ^1^H NMR (400 MHz, CDCl_3_): δ = 7.52 (s, 1H), 7.43–7.34 (m, 3H), 7.30–7.24 (m, 3H), 7.17–7.10 (m, 2H), 5.71 (s, 1H), 5.57 (d, *J* = 3.4 Hz, 2H), 4.15–4.13 (m, 1H), 3.93 (d, *J* = 12.0 Hz, 2H), 2.01–1.16 (m, 10H). ^13^C NMR (100 MHz, CDCl_3_): δ = 160.5 (d, *J* = 248.3 Hz), 154.2, 146.0, 135.4, 133.2, 130.9 (d, *J* = 8.3 Hz), 130.6 (d, *J* = 3.3 Hz), 129.9, 129.8, 129.3, 127.9, 124.8 (d, *J* = 3.8 Hz), 122.2, 121.8 (d, *J* = 14.7 Hz) 115.8 (d, *J* = 21.1 Hz), 57.9, 51.9, 47.6, 47.6, 42.3, 32.6, 32.5, 25.2, 25.1, 24.7. HRMS (ESI^+^): m/z: Calcd. for C_24_H_27_ClFN_8_ [M + H]^+^: 481.2031; Found: 481.2035.

*1-(1-(tert-butyl)-1H-tetrazol-5-yl)-N-((1-(2-fluorobenzyl)-1H-1,2,3-triazol-4-yl)methyl)-1-(4-methoxyphenyl)methanamine* (**13m**). Based on the GP1, propargylamine (23.2 µL, 0.36 mmol), 4-methoxybenzaldehyde (44.1 µL, 0.36 mmol), TMSN_3_ (57.2 µL, 0.43 mmol), cyclohexyl isocyanide (45.1 µL, 0.36 mmol), 2-fluorobenzyl bromide (43.7 µL, 0.36 mmol), sodium azide (28.2 mg, 0.43 mmol), CuSO_4_^.^5H_2_O (4.0 mg, 0.016 mmol), and sodium ascorbate (6.4 mg, 0.032 mmol) were used, and 13m was obtained as a yellow oil (yield 42.1 mg, 54%). R*_f_* = 0.40 (Hexane-AcOEt 4:6 v/v); ^1^H NMR (400 MHz, CDCl_3_): δ = 7.48 (s, 1H), 7.37–7.31 (m, 1H), 7.28–7.24 (m, 1H), 7.19–7.07 (m, 4H), 6.81 (d, *J* = 7.9 Hz, 2H), 5.54 (s, 2H), 5.40 (s, 1H), 3.87–3.75 (m, 5H), 1.52 (s, 9H). ^13^C NMR (100 MHz, CDCl_3_): δ = 160.5 (d, *J* = 248.1 Hz), 159.5, 155.5, 146.6, 130.9 (d, *J* = 8.1 Hz), 130.6 (d, *J* = 3.3 Hz), 130.3, 129.4, 124.8 (d, *J* = 3.7 Hz), 122.1, 121.8 (d, *J* = 14.2 Hz), 115.8 (d, *J* = 21.2 Hz), 114.2, 61.4, 56.6, 55.2, 47.7, 47.6, 42.1, 29.8. HRMS (ESI^+^): m/z: Calcd. for C_23_H_28_FN_8_O [M + H]^+^: 451.2370; Found: 451.2375.

*1-(1-cyclohexyl-1H-tetrazol-5-yl)-N-((1-(2-fluorobenzyl)-1H-1,2,3-triazol-4-yl)methyl)-1-(4-methoxyphenyl)methanamine* (**13n**). Based on the GP1, propargylamine (23.2 µL, 0.36 mmol), 4-methoxybenzaldehyde (44.1 µL, 0.36 mmol), TMSN_3_ (57.2 µL, 0.43 mmol), cyclohexyl isocyanide (45.1 µL, 0.36 mmol), 2-fluorobenzyl bromide (43.7 µL, 0.36 mmol), sodium azide (28.2 mg, 0.43 mmol), CuSO_4_^.^5H_2_O (4.0 mg, 0.016 mmol), and sodium ascorbate (6.4 mg, 0.032 mmol) were used, and 13n was obtained as a yellow oil (yield 48.8 mg, 28%). R*_f_* = 0.42 (Hexane-AcOEt 4:6 *v/v*); ^1^H NMR (400 MHz, CDCl_3_): δ = 7.49 (s, 1H), 7.37–7.35 (m, 1H), 7.30–7.24 (m, 3H), 7.17–7.09 (m, 2H), 6.86 (d, *J* = 8.7 Hz, 2H), 5.57 (s, 2H), 5.26 (s, 1H), 4.22–4.16 (m, 1H), 3.92–3.78 (m, 5H), 2.43 (bs, 1H), 1.90–1.17 (m, 10H). ^13^C NMR (100 MHz, CDCl_3_): δ = 160.5 (d, *J* = 248.1 Hz), 159.6, 154.6, 146.1, 130.9 (d, *J* = 8.2 Hz), 130.6 (d, *J* = 3.2 Hz), 129.6, 128.7, 124.8 (d, *J* = 3.6 Hz), 122.2, 121.8 (d, *J* = 14.5 Hz), 115.8 (d, *J* = 21.1 Hz), 114.3, 57.8, 55.7, 55.3, 47.7, 47.6, 41.9, 32.4, 32.4, 25.2, 25.2, 24.7. HRMS (ESI^+^): m/z: Calcd. for C_25_H_30_FN_8_O [M + H]^+^: 477.2527; Found: 477.2548.

*1-(1-(tert-butyl)-1H-tetrazol-5-yl)-N-((1-(2-fluorobenzyl)-1H-1,2,3-triazol-4-yl)methyl)-1-(2-fluorophenyl)methanamine* (**13o**). Based on the GP1, propargylamine (23.2 µL, 0.36 mmol), 2-fluorobenzaldehyde (38.2 µL, 0.36 mmol), TMSN_3_ (57.2 µL, 0.43 mmol), *tert*-butyl isocyanide (41.0 µL, 0.36 mmol), 2-fluorobenzyl bromide (43.7 µL, 0.36 mmol), sodium azide (28.2 mg, 0.43 mmol), CuSO_4_^.^5H_2_O (4.0 mg, 0.016 mmol), and sodium ascorbate (6.4 mg, 0.032 mmol) were used, and 13o was obtained as a beige solid (yield 48.4 mg, 31%). m.p. = 70–72 °C, R*_f_* = 0.53 (Hexane-AcOEt 4:6 *v/v*); ^1^H NMR (400 MHz, CDCl_3_): δ = 7.54 (s, 1H), 7.37–7.25 (m, 4H), 7.17–7.05 (m, 4H), 5.79 (s, 1H), 5.57 (s, 2H), 3.90 (d, *J* = 13.3 Hz, 2H), 1.55 (s, 9H). ^13^C NMR (100 MHz, CDCl_3_): δ = 160.5 (d, *J* = 248.0 Hz), 159.8 (d, *J* = 247.1 Hz), 154.5, 146.2, 130.8 (d, *J* = 8.1 Hz), 130.5 (d, *J* = 3.3 Hz), 130.2 (d, *J* = 8.3 Hz), 128.9 (d, *J* = 3.1 Hz), 125.7 (d, *J* = 14.1 Hz), 125.0 (d, *J* = 3.6 Hz), 124.7 (d, *J*=3.8 Hz), 122.1, 121.9 (d, *J* = 14.4 Hz), 115.8 (d, *J* = 12.0 Hz), 115.6 (d, *J* = 12.8 Hz), 61.5, 49.6, 49.5, 47.6, 47.6, 42.7, 29.6. HRMS (ESI^+^): m/z: Calcd. for C_22_H_25_F_2_N_8_ [M + H]^+^: 439.2170; Found: 439.2181.

### 3.2. Cell Line

The breast tumor cell line MCF-7 was grown in Dulbecco’s Modified Eagle’s Medium (DMEM) (Invitrogen Corporation, Carlsbad, CA, United States) enriched with 5% fetal bovine serum (FBS). Medium change and passage were performed every 3 and 4 days, respectively. The MCF-7 cell line was kindly provided by Ph.D. Victor Treviño from Tecnológico de Monterrey. 

### 3.3. Cell Proliferation Analysis 

Cell proliferation was quantified by violet crystal dye in 1× phosphate-buffered saline (PBS) (2.7 mM KCl, 1.8 mM KH_2_PO_4_, 136 mM NaCl, 10 mM Na_2_HPO_4_ pH 7.4). The treated cells were incubated in methanol for 15 min and washed two times with water. Cells were dyed with 0.1% crystal violet and washed three times with water, and finally, violet crystal was recovered with 10% acid acetic to be analyzed in microplate reader Multiskan GO Spectrophotometer (Thermo Scientific™, Ratastic, Finland).

## 4. Conclusions

Six new bonds (one C-C, four C-N, and one N-N) were formed in the one-pot reaction under a cascade process consisting of three reaction steps: Ugi-azide reaction, S_N_2, and CuAAC reaction, which proceeded smoothly. The synthetized hybrid compounds, particularly **13f** and **13l**, exhibited a moderate cell proliferation inhibition degree with a remarkable effect on the MCF-7 cells. These later compounds have the cyclohexyl group and halogens atoms in their structures. Structural modification of these compounds may increase their activity in further cytotoxicity assays. 

## Figures and Tables

**Table 1:**
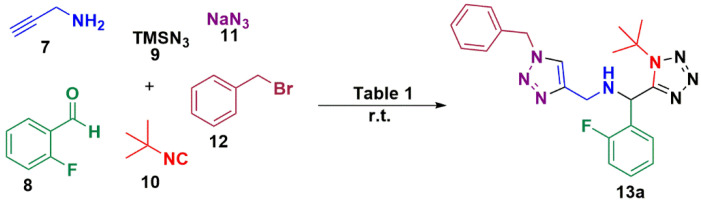
Optimization of the one-pot six-component reaction conditions.

Entry	Solvent	Yield %
1	MeOH [1M]	58 ^1^
2	Glycerol [1M]	11 ^1^
3	*t*-BuOH:H_2_O [0.1M, 1:1 *v*/*v*]	15 ^1^
4	TFE [1M]	ND ^2^

^1^ Isolated yield.^2^ ND = not detected.

**Table 2:** IC_50_ of the compounds **13a** to **13o** in MCF-7 cells.

Product	IC_50_(µM) MCF-7	Product	IC_50_(µM) MCF-7
**13a**	200	**13i**	200
**13b**	31.63	**13j**	44.51
**13c**	200	**13k**	60.77
**13d**	55.48	**13l**	22.84
**13e**	62.73	**13m**	200
**13f**	19.76	**13n**	29.25
**13g**	200	**13o**	200
**13h**	41.67		

## Data Availability

Not applicable.

## Supplementary Materials

The Supplementary Materials are available online.

## Sample Availability

Samples of the compounds **13a-o** are available from the authors.
